# Outcomes for Early Years’ Children in Contact with Social Services in Northern Ireland: A Data Linkage Study Protocol

**DOI:** 10.23889/ijpds.v11i1.3401

**Published:** 2026-05-19

**Authors:** Rachel Leonard, Lisa Bunting

**Affiliations:** 1 School of Social Sciences, Education and Social Work, Queen’s University Belfast, United Kingdom

**Keywords:** Children’s social care, Administrative data, Data linkage, Northern Ireland, Maternity Care, Protocol

## Abstract

**Introduction:**

Children’s social care involvement in the early years has significant implications for child development and family wellbeing. While previous research has highlighted associations between deprivation and child welfare interventions, there remains limited understanding of the timing, recurrence, and intergenerational patterns of social care contact. This study addresses these gaps using linked administrative data in Northern Ireland.

**Objective:**

To examine the rates and predictors of antenatal, postnatal and early years (0-2 years) involvement with child and family social work and repeat removals of children from the same mother.

**Method:**

A population-level retrospective cohort study will be conducted using linked data from the Northern Ireland Maternity System (NIMATS) and the Social Services Client Administration and Retrieval Environment (SOSCARE). Mothers with first births between 2010–2017 will form the primary cohort. Variables of interest include maternal and child demographics and deprivation. Outcomes included referral, investigation, child protection registration (CPR), and looked after child (LAC) status. Analyses will include descriptive statistics, Cox regression for time-to-event outcomes, and logistic regression for binary outcomes. Subgroup analyses will examine repeat contact and care-experienced mothers.

**Results:**

Results from this study will describe the characteristics of those infants and mothers who are involved in social services in Northern Ireland, at different thresholds of involvement, and their outcomes following that involvement.

**Conclusions:**

This study will add to the evidence on early identification and sustained support for at-risk mothers. Linked administrative data offers valuable insights into system dynamics and can inform targeted interventions and policy reform aimed at reducing inequalities and improving outcomes for families.

## Introduction

Over the past decade in the UK there has been increasing interest in using children’s social care data to better understand the factors which drive child welfare intervention rates e.g. children designated as ‘in need’; those subject to child protection processes, and those admitted into state care. Despite recent advances in our understanding of the factors driving referrals to child and family social work and subsequent child welfare interventions, there are still significant limitations to the current evidence [1–8]. Previous research has shown the link between individual and area level deprivation and increased risk of contact with child protection and looked after systems [1–5]. Equally, various international and UK research have shown associations between reductions in social welfare payments, increases in unemployment and child poverty and increases in contact with these systems [6–8].

Recent research has highlighted increasing trends in newborn and very young children entering child welfare processes and care proceedings in England and Scotland [9–11]. Similarly, previous research by the authors, also highlighted widening inequality over time in the rates of children becoming looked after in NI, a trend largely driven by increasing numbers of children aged 0-4 years taken into care [5, 12]. Understanding these rates is particularly important in the early years [13–15]. Early childhood represents a critical period of rapid cognitive, emotional, and social development, during which experiences exert a profound and lasting influence on life trajectories [13, 15]. Adversity during this stage can disrupt developmental processes and increase the risk of long-term negative outcomes [13, 15]. Examining social service engagement during this period provides crucial insight into both the prevention of harm and interventions aimed at improving developmental outcomes across the life course [14].

Furthermore, repeat or successive removals of children from birth parents, following birth or early in the postnatal period has also been identified as a significant concern. Previous research using English administrative data has shown that the family court system reprocesses a sizeable percentage of women (24%) through repeat episodes of care proceedings, with young women aged sixteen to nineteen years most at risk of recurrence [16]. In addition, the majority of repeat clients, return within a short space of time (median interval is seventeen months), typically following the birth of a new infant [16]. Further emphasising the importance of understanding social service involvement in this critical time in a child’s life. While producing important findings, this analysis focused on care proceedings only, missing the scope of social work involvement prior to reaching the stage of formal proceedings. A referral to social services may indicate emerging concerns or requests for support, while a child protection investigation signals suspected harm or significant risk, while entry into out-of-home care represents the most intensive statutory intervention. Distinguishing between these levels of contact is important because they capture distinct differences of adversity, surveillance, and service provision, each of which may carry distinct developmental implications for infants and children.

This study will address these gaps as well as introducing a much wider range of child and family characteristics, together with area level deprivation, into analysis to address the research questions:

1. *What are the rates of, and factors associated with, antenatal, postnatal and early years (0-2 years) involvement with child and family social work in the general population in Northern Ireland?*
2. *What are the rates of, and factors associated with repeat removals of multiple children from the same mother and subsequent care outcomes?*

## Methods

### Study Design

A population-level retrospective cohort study will be conducted. The data requested will provide access to individual child level data which records child contact with child and family social work services, through the Social Services Client Administration and Retrieval Environment (SOSCARE). As well as data linkage to area level indicators of deprivation (postcode linkage to NI Multiple Deprivation Measures through the Central Postcode Directory). While the primary time period for study is 2010-2017, SOSCARE data for the years 1990-2024 will be requested to allow for the identification of previous contact with social service prior to the study period. The data will be linked with the Northern Ireland Regional Maternity System (NIMATS) through a unique identifier: the Health and Social Care Number (HCN). NIMATS provides a range of demographic and clinical information on mothers and infants and captures data relating to the current complete maternity process, as well as details about the mother’s past medical and obstetric history. It is a key source for data on birth numbers, interventions, maternal risk factors, birth weights, maternal smoking, and other lifestyle factors. As such, data linkage between NIMATS and SOSCARE will provide additional demographic and risk factor variables relating to the mother, infant, family situation to SOSCARE analysis and allow for comparison of mother and children known to social service and those who have never had contact. NIMATS data for the years 2010-2024 will be requested, as all hospitals have been using NIMATS from 2010 onwards.

### Study Population and Eligibility Criteria

The study population will include all births that took place in Northern Ireland between 2010 and 2017. While a wider timeframe of data will be requested, the time period for analysis will be based on the availability and accuracy of both datasets. For example, NIMATS became consistently used across Northern Ireland from 2010 onwards [17], whereas SOSCARE was phased out of use across some of the health and social care trusts (HSCT) from 2017 onwards [18]. This has resulted in partial geographical coverage of SOSCARE data, as three of the five HSCTs in Northern Ireland migrated to a new information management system (PARIS) (18). For comparisons of those who had contact with social services versus those who do not, we will include all births (mothers and infants). We will determine “contact” as the mothers of infants who had a first referral between 2010-2017. For comparison of levels of contact, we will include infants who had a referral to social services, child protection investigation, or became looked after between 2010 – 2017. For repeat removals, we will include mothers who have had repeat removals of children from 2010 onwards. Due to limitations with the SOSCARE dataset we are not able to accurately link mothers to any children that may be in the SOSCARE dataset prior to 2010 (those with prior social service involvement). However, we will be able to see where a mother herself has been in care (i.e. looked after) and will use this as a co-variate for further analysis.

### Datasets and Variables of Interest

The two datasets that will be used within this study are presented in Table 1. Both datasets derive from Northern Ireland’s Health and Social Care system. Health and Social Care is the publicly funded healthcare system in Northern Ireland, which is part of the United Kingdom’s National Health Service (NHS). Social services and maternity care are delivered through five regional HSCTs, with each HSCT collecting administrative data [18]. The NIMATS captures data on all pregnant women and their infants born in NI (17). While SOSCARE captures data on all children involved in social care in NI [18]. Both the NIMATS and SOSCARE contain a unique health and social care number (HCN) for both mother and infant. We will link individual infant and mother records using these HCN’s in both datasets. Variables of interest relate to 1) maternal demographics, 2) levels of deprivation, 3) levels of social work involvement, and 4) social work involvement outcomes for the infant and mother.

**Table 1:** Datasets Used Within the Study

**Dataset**	**Brief description**
Northern Ireland Regional Maternity System (NIMATS)	Includes data regarding the mother and baby from booking (antenatal), child birth and postnatal stages for all births in Northern Ireland [17].
Social Services Client Administration and Retrieval Environment (SOSCARE)	Includes data on all children’s social care activity which includes referrals, Children in Need, Child Protection Investigations, Child Protection Registrations and Looked After Children [18].

Maternal demographic data includes the following:

- Maternal age (years)
- Marital status (Married, single, partner, divorced/separated)
- Smoking (yes/no)
- Alcohol (yes/no)
- Mental health (yes (any antenatal, postnatal or pre existing mental health)/no)
- Domestic violence (no disclosure/ historical disclosure/ current disclosure)
- Partner status (yes/no)
- Mothers care-experienced status (yes/no)
- Health and Social Care Trust (HSCT)
- Parity (number of live births >24 weeks)

Child demographic data includes:

- Birth year
- Birth weight (grams)
- APGAR (Appearance, Pulse, Grimace, Activity, and Respiration) score (1 – 10)
- Disability (yes/no)

The data relating to deprivation includes the following;

- Area level deprivation decile (1 (most deprived) – 10 (least deprived): based on area at time of antenatal booking appointment)
- The Northern Ireland Multiple Deprivation Measure 2017 (ranking 890 Super Output areas (SOAs) in Northern Ireland from the most deprived (rank 1) to the least deprived (rank 890) (based on area at time of antenatal booking appointment).

Outcome data on social work involvement will include the following;

- Referral to social services (yes/no)
- Child protection investigation (yes/no)
- Registered to child protection register (yes/no)
- Looked after (yes/no)

### Data Analysis

Following an exploration of the data, we will examine the overall quality and completeness of the data and each variable. Frequency tables will be used to identify any outliers and will be reviewed on a case by case basis, either being corrected or removed. Duplicate records within each dataset will be examined (for example, where an infant_ID is present more than once in the dataset) and either corrected or removed. For variables related to maternal and child demographics, and deprivation level, the NIMATS dataset will be prioritised given it overall reliability. For variables related to social work involvement and social work outcomes, the SOSCARE dataset will be prioritised.

Data analysis will be conducted across three strands—population-level cohort analysis, repeat contact, and intergenerational transmission. The unit of analysis will be the mother.

1. **Population-Level Cohort Analysis**
  We will employ both descriptive and inferential statistics to examine the timing, risk, and recurrence of social care contact following childbirth.
  - **Descriptive Statistics**: Proportions and rates of social care involvement (referral, investigation, child protection registration [CPR], and looked after child [LAC]) will be calculated for mothers following their first and second births.
  - **Survival Analysis**: Cox proportional hazards models for each outcome will estimate time-to-event outcomes (e.g., time from birth to first referral, investigation, CPR or LAC involvement). Separate models will be fitted the four outcomes, with predictors including all maternal and child demographics, deprivation (detailed above).
  - **Sensitivity Analyses**: Subgroup analyses will be conducted using parity-complete mothers and Trust area coverage, and by restricting the cohort to specific birth years (e.g., 2010–2015).
2. **Repeat Contact Analysis**
  This strand of analysis will focus on identifying patterns and predictors of repeated social care involvement.
  - **Descriptive Profiles**: Frequencies and cross-tabulations will be used to compare mothers with single versus repeat contact episodes, including timing between referrals and escalation patterns.
  - **Logistic Regression**: Binary models will be used to predict repeat contact (same child or multiple children) and statutory involvement, using maternal and child level predictors (detailed above).
  - **Subgroup Comparisons**: Analyses will distinguish between limited contact (e.g., referral only) and statutory involvement (e.g., CPR or LAC).
3. **Intergenerational Analysis**
  This component will examine the relationship between mothers’ own care histories and their subsequent involvement with social care as parents.
  - **Descriptive and Bivariate Analysis**: Care-experienced mothers will be profiled based on looked after data (i.e. if mothers are present in data from 1990 onwards) comparing those with and without social care contact post-birth.
  - **Regression Models**: Binary logistic regression will be used to identify predictors of social care contact among care-experienced mothers. Key variables included age at first care episode, placement type (e.g. kinship, residential), duration, and maternal demographics (detailed above).

### Data Management and Ethics

This study was not required to obtain internal university ethical approval, as per university policy on the use of secondary data. Instead, permission and governance is obtained through the HSC Honest Broker Service’s (HBS), robust structures for the access of administrative data.

All data access and linkage will be conducted and facilitated through the HSC HBS, which is the accredited Secure Data Environment for administrative data in NI. All researchers accessing data through the HBS are required to obtain Accredited Safe Researcher status, as part of the service’s ethics procedures, which both authors have obtained. Following project approval, the HBS will extract and de-identifies both requested datasets, removing direct identifiers before making data available. Researchers will not receive or handle raw identifiable information at any stage of the project. Data will be accessed and analysed exclusively within the HBS Secure e-Research Platform (UK SeRP), a secure virtual desktop environment.

## Results

Following this study, we will describe the characteristics of those infants and mothers who are involved in social services in Northern Ireland, at different thresholds of involvement, and their outcomes following that involvement, compared to those infants and mothers who had no social work input. We will discuss the significance of the results, highlighting any disparity between groups, with a sub-comparison of care-experienced mothers. We will also report on the availability and usefulness of the current data on mothers and infants involved in social services in both the NIMATS and SOSCARE datasets, highlighting any benefits and limitations.

## Discussion

This study will provide a comprehensive population-level analysis of mothers’ contact with children’s social care services in Northern Ireland, using linked administrative data across maternity and social care systems. By examining both first and second births and incorporating intergenerational and repeat contacts, the findings will offer new insights into the timing, recurrence, and predictors of social care involvement across the spectrum of intervention.

The use of longitudinal linkage between NIMATS and SOSCARE datasets will enable the identification of key maternal and child level risk factors associated with social care contact, including sociodemographic disadvantage, maternal health indicators, and prior involvement. Consistent with previous research (2, 8), this study will provide further understanding of the association between deprivation and increased likelihood of statutory intervention. The inclusion of birth spacing and prior contact as predictors in models will add further nuance to our understanding of recurrence risk, particularly around repeat removals and care-experienced mothers (13).

Importantly, the study will examine the elevated risk of early contact—particularly in the antenatal and postnatal periods—among certain subgroups, including care-experienced mothers. This will further add to prior evidence around intergenerational transmission of social care involvement (5) and can aid in the development of targeted early intervention strategies. The ability to quantify recurrence and escalation patterns across birth episodes will provide a valuable evidence base for service planning and prevention efforts.

While this study will utilise data within Northern Ireland, several features of the findings are likely to have relevance beyond this specific context. Many high-income countries operate tiered child welfare and social service systems that progress from referral and assessment to more intensive statutory intervention, reflecting similar underlying processes of risk identification and response. The developmental mechanisms linking early adversity, service involvement, and later outcomes are also well established and are not unique to Northern Ireland. Nevertheless, differences in legal frameworks, thresholds for intervention, service availability, and socioeconomic contexts may influence both patterns of contact and their consequences. Future research using comparable administrative data in different national and international contexts would help to assess the extent to which these findings generalise.

## Strengths and Limitations

From a methodological perspective, the study will consider the feasibility and utility of linking maternity and social care datasets to generate population-level insights. This study will enable population-level analysis of early childhood social service involvement with minimal loss to follow-up. The longitudinal linkage also permits assessment of timing and intensity of involvement during a developmentally critical period.

However, the proposed study presents some limitations. The reliance on administrative data means that certain contextual factors—such as informal support networks or unrecorded service use—will not be captured. In addition, observed associations may be influenced by confounding and residual confounding despite adjustment for measured covariates. While the study includes mothers with multiple births, it excludes those whose children were removed prior to the study period, potentially underestimating recurrence rates. Finally, administrative data reflect service contact rather than underlying need, meaning findings may be shaped by thresholds for intervention and institutional practices.

Despite these limitations, the study contributes to a growing body of evidence on child welfare inequalities and social services interventions. It offers actionable insights for policymakers, practitioners, and researchers, particularly in the context of ongoing reforms to children’s services in Northern Ireland.

## Conclusion

This study will provide a robust population-level analysis of maternal contact with children’s social care services in Northern Ireland, drawing on linked administrative data to explore patterns of involvement across birth episodes and generations. By integrating maternity and social care datasets, we will be able to identify key risk factors, timing of first contact, and recurrence of involvement, providing a more nuanced understanding of social service intervention and family trajectories.

## Data Availability

SOSCARE data are available for research projects in the public interest that relate to Health and Social Care, subject to application and approval by the Honest Broker Service Governance Board (for information contact honestbrokerservice@hscni.net).

## Abbreviations

SOSCARE:	Social Services Client Administration and Retrieval Environment
NIMATS:	Northern Ireland Regional Maternity System
HCN:	Health and Care Number
HSCT:	Health and Social Care Trust
CPR:	Child Protection Register
LAC:	Looked After Child
